# The role of childbirth educators in the context of the COVID-19 pandemic

**DOI:** 10.4069/kjwhn.2022.02.25

**Published:** 2022-03-17

**Authors:** Hyun Kyoung Kim

**Affiliations:** Department of Nursing, Kongju National University, Gongju, Korea

Childbirth education helps to deliver information regarding health care to pregnant women and their families during the antenatal and postnatal periods. Culturally, Korea has a unique type of childbirth education, termed *taegyo*, which helps in maternal health care and stimulates the cognitive development of the fetus in the womb [1]. In modern society, this tradition continues to be taught in prenatal classes for pregnant women and their families, mainly at hospitals and public health care centers. Prenatal classes play a role in encouraging maternal physical, psychological, and social health through self-care during pregnancy. These classes deliver information on a wide-ranging and deep understanding of the birth process and readiness for the maternal role, and they play a valuable role as a mode for evidence-based nursing care to be shared with pregnant women. In addition to learning about diet, nutrition, vaccination, exercise, rest, activity, the birth process, pain control during labor, breastfeeding, and practical approaches to daily activities, pregnant women gain emotional benefits through communication between educators and pregnant women. Childbirth education enhances parental attachment, motherhood, confidence, and childbearing efficacy, and it relieves psychological distress and the postpartum blues [2].

Although birth education is important for pregnant women, the coronavirus disease 2019 (COVID-19) pandemic changed the social atmosphere and culture, especially in the field of health-related education [3]. Face-to-face education was curtailed in Korea because of social distancing and quarantine. Birth education has also been limited in order to avoid personal contact in the past 2 years since COVID-19. Pregnant women have lost educational opportunities to obtain knowledge, have appropriate attitudes reinforced, and develop the necessary skills for healthy pregnancy and birth because many birth classes have been shut down. Even when a birth class is open, only a limited number of pregnant women can participate in on-site education because of governmental quarantine rules. The strengthened social distancing regulations to prevent the spread of COVID-19 permit private gatherings of only up to four people nationwide in Korea [4]. Gradually, midwives, nursing educators, and medical staff have tried to provide remote birth education.

However, this leads to an important question: can remote birth education be an acceptable substitute for face-to-face birth classes? Some insights into this question are offered by recent studies, such as a systematic review and meta-analysis of nine randomized controlled articles on internet-based prenatal education interventions, which found interventions delivered via online reduced maternal depression [5]. Internet-based education is defined as the delivery of organized educational content between educators and learners using computer networks, and it is characterized by interactive communication, self-learning, and tutoring. Remote education, e-learning, and distance education are used as types of internet-based education to substitute for face-to-face learning as needed. Terminology on such modes of communicating information includes internet-based education, online education, mHealth, teleHealth, social networking services, social messaging services, kiosks, and animations [6]. Virtual reality, augmented reality, mixed reality, and game-based intervention, collectively termed the “metaverse,” have emerged as educational techniques to immerse learners in educational content [7]. A game-based decision aid for prenatal education has also been found to be effective for enhancing prenatal screening [7].

The advantage of these modalities for the educator is that they are cost-effective and have a high ripple effect since the content is delivered to a large number of people. These methods can deliver standardized and high-quality education [5]. From the learner’s point of view, visual information such as photos and videos can be more easily understood than text information. Learners showed high levels of understanding, acceptance, adaptability, and feasibility [6]. In addition, learners do not need transportation or child care, can use these materials at a convenient time according to their schedule, and can save time [6]. Therefore, internet-based education, which has advantages in terms of time and space, can be an effective alternative in the COVID-19 situation, which has necessitated social distancing.

However, efforts to implement internet-based education should aim to overcome the unintended inequalities of technical development, which have been characterized as a double-edged sword. Firstly, smart devices are not common equipment and there are financial and local disparities in internet access. Childbirth educators should pay careful attention to vulnerable pregnant women when they plan birth education via an internet-based intervention. Internet literacy should be considered and internet-literacy education may have to precede birth education. Policymakers can narrow the gaps of information disparities in the context of the pandemic era [3]. Therefore, childbirth educators can make proposals to policymakers and stakeholders regarding ways of preparing educational techniques that involve the delivery of information through the internet. In particular, these efforts should carefully consider susceptible and minor pregnant women (including those with limited internet literacy), pregnant women from multicultural backgrounds, socioeconomically vulnerable women, and pregnant women who have illnesses, with the goal of ensuring that they can benefit from these new types of interventions.

Secondly, the quality of internet-based education can be improved and strengthened through active participation of childbirth educators, who encourage pregnant women through social support. Face-to-face education includes communication and natural social chemistry between learners and educators. However, it is easy for internet-based interventions to lack such supportive interactions. Therefore, childbirth educators should actively engage with and support pregnant women via social networking services, offering counseling, coaching, and question-and-answer sessions during education. Given that the pandemic situation has limited pregnant women to remain at home and has affected their mood [8], childbirth educators should place a particular emphasis on caring for maternal mental health, such as depression, anxiety, and other aspects of psychological wellbeing.

Lastly, pregnant women are often worried both about COVID-19 infection and the negative effects of vaccination, with the goal of avoiding the risk of harm to fetal health. The low acceptance rate of COVID-19 vaccination and the possibility of the low planning for parenthood in the last 2 years have underscored the importance of unanswered questions regarding safety for maternal and fetal health [9]. A recent study in which 539 pregnant women participated found no significant differences in the rates of short-term side effects after vaccination [10]. However, more evidence is necessary for the long-term outcome of maternal and infant health, including the breastfeeding and postpartum periods. Childbirth educators play a role in providing cutting-edge knowledge about the pandemic and research about maternal knowledge, beliefs, attitudes, values, and skills of self-care during pregnancy in the context of the COVID-19 pandemic. This suggests that childbirth educators exert a special influence on maternal and infant health through their valuable support.
